# Investigation of Oncogenic Cooperation in Simple Liver-Specific Transgenic Mouse Models Using Noninvasive *In Vivo* Imaging

**DOI:** 10.1371/journal.pone.0059869

**Published:** 2013-03-28

**Authors:** Hye-Lim Ju, Sang Hoon Ahn, Do Young Kim, Sinhwa Baek, Sook In Chung, Jinsil Seong, Kwang-Hyub Han, Simon Weonsang Ro

**Affiliations:** 1 Liver Cirrhosis Clinical Research Center, Yonsei University College of Medicine, Seoul, Korea; 2 Brain Korea 21 Project for Medical Science College of Medicine, Yonsei University, Seoul, Korea; 3 Department of Internal Medicine, Yonsei University College of Medicine, Seoul, Korea; 4 Department of Radiation Oncology, Yonsei University College of Medicine, Seoul, Korea; University of North Carolina School of Medicine, United States of America

## Abstract

Liver cancer is a complex multistep process requiring genetic alterations in multiple proto-oncogenes and tumor suppressor genes. Although hundreds of genes are known to play roles in hepatocarcinogenesis, oncogenic collaboration among these genes is still largely unknown. Here, we report a simple methodology by which oncogenic cooperation between cancer-related genes can be efficiently investigated in the liver. We developed various non-germline transgenic mouse models using hydrodynamics-based transfection which express HrasG12V, SmoM2, and a short-hairpin RNA down-regulating p53 (shp53) individually or in combination in the liver. In this transgenic system, firefly luciferase was co-expressed with the oncogenes as a reporter, allowing tumor growth in the liver to be monitored over time without an invasive procedure. Very strong bioluminescence imaging (BLI) signals were observed at 4 weeks post-hydrodynamic injection (PHI) in mice co-expressing HrasG12V and shp53, while only background signals were detected in other double or single transgenic groups until 30 weeks PHI. Consistent with the BLI data, tumors were observed in the HrasG12V plus shp53 group at 4 weeks PHI, while other transgenic groups failed to exhibit a hyperplastic nodule at 30 weeks PHI. In the HrasG12V plus shp53 transgenic group, BLI signals were well-correlated with actual tumor growth in the liver, confirming the versatility of BLI-based monitoring of tumor growth in this organ. The methodology described here is expected to accelerate and facilitate *in vivo* studies of the hepatocarcinogenic potential of cancer-related genes by means of oncogenic cooperation.

## Introduction

Liver cancer is a complex multistep process that involves genetic alterations in multiple proto-oncogenes and tumor suppressor genes [1]–[3]. Hundreds of genes are known to play roles in the initiation and progression of hepatocellular carcinoma (HCC) after having undergone a genetic alteration [3], [4]. An oncogenic mutation in a single gene, however, usually fails to induce liver cancer, as shown in transgenic mouse models expressing single oncogenes [5], [6]. This implies that oncogenic collaboration among multiple cancer-related genes is required to induce HCC. Identifying oncogenes that cooperatively induce HCC will facilitate a greater understanding of the genetic mechanism(s) underlying liver carcinogenesis and will provide new insights into the genetic pathway that leads to HCC.

Ras proteins are the prototype 21-kDa GTPases and serve as master regulators in a myriad of signaling cascades. An activating mutation in *ras* genes (a mutation at codon 12 resulting in the substitution of valine for glycine, for example) leads to constitutive activation of the Ras signaling pathways. An activation of the Ras signaling pathways is found in more than 50% of HCCs [7], [8]. Another pathway that is frequently activated in HCC is the hedgehog signaling pathway, which is closely related to cell cycle, proliferation, and angiogenesis. An activating mutation in Smo (such as a mutation at codon 535 leading to substitution of leucine for tryptophan) causes hedgehog signaling to be constitutively active and is found in a variety of tumors [9], [10]. The p53 pathway is a major tumor-suppressing signaling pathway that limits cell survival and induces cell-cycle arrest. Loss of p53 function is frequently found in tumors of diverse cellular origins, including HCC, and is considered a critical step in tumor development [1], [11].

To better understand the roles of genetics in HCC development, genetically modified mouse (GEM) models in which expression of a specific oncogene or tumor suppressor gene is manipulated have been developed [5], [6]. The development of a GEM model, however, usually involves expensive and time-consuming processes, thus generation of a variety of GEM models is highly challenging.

Non-germline GEM models utilize transfection or transduction of specific target tissues with vectors expressing a gene of interest [12]. This approach can significantly reduce the time and resources needed to generate transgenic models and is thus suitable for testing the biological functions of various genes in a relatively short time period *in vivo*. Applying the non-germline transgenic approach, Zender *et al*. developed transgenic models of HCC by *ex vivo* transduction of embryonic hepatoblasts with retroviruses expressing various oncogenes, followed by transplantation of the transduced hepatoblasts into the liver [13]. In another very elegant and simple method, naked DNA plasmids encoding a gene of interest are directly delivered into the liver by hydrodynamics-based transfection [14]. For stable gene expression, the transfection method has been coupled with the *Sleeping Beauty* transposon system, which mediates chromosomal integration of a transgene [15]–[17]. In this transgenic system, the expression cassette of a transgene is placed between two inverted terminal repeats (IRs), rendering the expression cassette transposable by *Sleeping Beauty* transposase. To achieve stable expression of a transgene, the transposon plasmids are hydrodynamically injected together with plasmids expressing *Sleeping Beauty* transposase, which excises the DNA regions flanked by the IRs and subsequently transfers them to chromosomes.

One methodological challenge in studies using animal models of HCC is that liver tumors are hard to observe due to the limitations in access to the organ and *in vivo* imaging techniques. Genes encoding fluorescent proteins or luciferases have been used to label tumor cells in xenograft cancer models, allowing growth of transplanted tumors to be observed by *in vivo* fluorescence or bioluminescence imaging (BLI) [18], [19]. The reporter genes have also been used in GEM models for tumor imaging; however, the additional genetic manipulation involved in expression of a reporter gene makes the use of a reporter unattractive.

Here, we report a method by which oncogenic collaboration of various cancer-related genes in the liver can be easily investigated *in vivo* by BLI of tumors. Using firefly luciferase as a reporter, tumor growth in the liver induced by a combination of oncogenes was successfully monitored over time without an invasive procedure.

## Materials and Methods

### Plasmids

The plasmid pCX-EGFP, which encodes enhanced green fluorescent protein (EGFP), was a generous gift from Dr. Masaru Okabe [20]. The whole expression cassette containing the chicken β-actin promoter, EGFP cDNA, and a rabbit β-globin polyadenylation signal was cloned into pT2/BH (a kind gift from Dr. Perry Hackett [21]), which has multiple cloning sites flanked by the IRs. The resulting plasmid is referred to as pT2/EGFP. A cDNA encoding an activated form of human Hras (HrasG12V) was PCR amplified from pBABE puro H-Ras V12 (plasmid #9051; Addgene) as a template using the following primer pair: forward, 5′-TTG AAT TCG CCA CCA TGA CGG AAT ATA AGC TGG TGG TGG-3′; and reverse, 5′-TTG AAT TCT TAG GAG AGC ACA CAC TTG C-3′. The amplified products were then digested with *Eco*RI and cloned into pT2/EGFP following digestion of the plasmid with the same restriction enzyme to remove the EGFP cDNA. The resulting plasmid is referred to as pT2/HrasG12V. A cDNA encoding an activated form of Smo was PCR amplified from pRK-SmoM2 as a template using the following primer pair: forward, 5′-TTG AAT TCG CCA CCA TGG CCG CTG GCC GCC CCG TG-3′; and reverse, 5′-TTG AAT TCT TAG AAG TCC GAG TCT GCA TC-3′. The amplified products were cloned into pT2/EGFP using the es described above. The resulting plasmid is referred to as pT2/SmoM2. pT2/shp53/GFP4, a transposon vector encoding a short hairpin RNA against tumor suppressor p53, was a gift from Dr. John Ohlfest and is hereafter referred to as pT2/shp53 [22]. The plasmid PT2/C-Luc//PGK-SB13, which encodes *Sleeping Beauty* transposase under the control of the phosphoglycerate kinase (PGK) promoter and harbors a transposon expressing firefly luciferase, was a kind gift from Dr. John Ohlfest.

### Transfection, Western Blotting, and Gli-induced Firefly Luciferase Expression Assays

NIH3T3 cells (CRL-1658; ATCC, Manassas, USA) were transiently transfected with 2 µg of DNA using FuGENE HD (Promega) according to the manufacturer’s instructions. For detection of downstream Ras pathway molecules, cells were harvested at 2 days post-transfection and lysed in 1× RIPA buffer (#9806; Cell Signaling). To evaluate the down-regulation of p53 expression by pT2/shp53, cells were transfected with pT2/EGFP (as a control) and pT2/shp53, and were irradiated with UVC (10 mJ/cm^2^) at 24 h post-transfection using an XL-1500 UV-crosslinker (Spectronics Corporation). Cells were lysed in 1× RIPA buffer at 8 h post-irradiation. Western blotting experiments were performed using standard methods. The following primary antibodies were purchased from Cell Signaling Technology: anti-p53 (#2524), anti-Akt (#9272), anti-phospho-Akt (#4060), anti-ERK1/2 (#9102), anti-phospho-ERK1/2 (#4370), anti-MEK1/2 (#9126), and anti-phospho-MEK1/2 (#9154). For internal controls, an anti-β-actin antibody (sc-47778, Santa Cruz) was used. Anti-mouse IgG HRP (sc-2005, Santa Cruz) and anti-rabbit IgG HRP (A0545, Sigma) were used as secondary antibodies. To evaluate activation of hedgehog signaling by pT2/SmoM2, cells were transfected with DNA mixtures containing 1 µg of pT2/SmoM2, 100 ng of a plasmid encoding *Renilla* luciferase (as a reference), and 1 µg of reporter plasmid (8×3′Gli-BS Luc carrying binding sites for Gli transcription factors, a gift from Dr. Hiroshi Sasaki) [23]. As a control, cells were transfected with DNA mixtures containing 1 µg of pT2/EGFP, 100 ng of a plasmid encoding *Renilla* luciferase, and 1 µg of the reporter plasmid. Luciferase activity was measured with the dual luciferase reporter assay system (Promega) according to the manufacturer’s instructions.

### Animals

All experiments using live mice were performed in strict accordance with the Guidelines and Regulations for the Care and Use of Laboratory Animals in AAALAC-accredited facilities, and were approved by the Animal Policy and Welfare Committee of the Yonsei University College of Medicine (Permit number: 2011-0250). Male 5- to 6-week-old C57BL/6 mice were purchased from Orientbio (Seongnam, Korea).

### Hydrodynamic Injection

Hydrodynamic injection was performed as described previously [14]. The plasmids pT2/HrasG12V, pT2/SmoM2, pT2/shp53, and PT2/C-Luc//PGK-SB13 were prepared with endotoxin-free Maxi Kits (Qiagen, Hilden, Germany). For the generation of HrasG12V transgenic mice, 25 µg of pT2/HrasG12V (6.6 kb) was mixed with 18.7 µg of PT2/C-Luc//PGK-SB13 (9.8 kb) such that the molar ratio of transposon plasmids expressing an oncogene to transposase-encoding vector was 2∶1. The plasmid pT2/HrasG12V was used as the molar standard for transposons. Mice of the same body weight each received the same molar amount of transposons, regardless of the types of transposons. For double transgenic groups, half-molar amounts of transposons for each transgene were mixed together; *i*.*e*., 12.5 µg of pT2/HrasG12V and 16 µg of pT2/SmoM2 (8.4 kb) were used to generate HrasG12V plus SmoM2 double transgenic mice. After mixing transposons with the transposase-encoding plasmids, DNA was suspended in 2 ml of lactated Ringer’s solution and was then injected into the lateral tail veins of male 6- to 7-week-old C57BL/6 mice (0.1 ml/g body weight) in less than 7 sec.

### Bioluminescence Imaging

The abdominal area of skin was depilated using a depilatory cream 1 day before imaging. On the day of imaging, mice were intraperitoneally injected with D-luciferin (150 mg/kg) and were placed in a light-tight mouse imaging chamber following anesthesia. A photographic (gray-scale) reference image was obtained at 10 min after the administration of D-luciferin and bioluminescence images were captured immediately thereafter. Images were obtained with a CCD camera cooled to −90°C, using the IVIS Imaging System (Caliper Life Sciences, Alameda, CA, USA). Regions of interest were drawn in the abdominal area and total counts (photons) in all areas were summed. The signal intensities of each defined region of interest were quantified as photon count rate per unit body area per unit solid angle subtended by the detector (photons/s/cm^2^/steradian).

### Liver Harvesting, Tissue Processing, and H&E Staining

After euthanizing mice, their livers were removed and rinsed in PBS. Samples collected from the livers were fixed overnight in freshly prepared neutral-buffered formalin. Fixed tissue samples were embedded in paraffin. Five-micron sections were placed on slides and stained with hematoxylin and eosin (H&E) in order to observe cell morphology. For immunofluorescence, unfixed tissues were embedded in OCT and were then frozen.

### Immunofluorescence

OCT-embedded frozen tissues were sectioned to a thickness of 5 µm using a cryostat microtome (Microm HM525; Thermo Fisher Scientific, Walldorf, Germany). Each section was placed on a slide and was subsequently fixed in acetone. Slides were washed with phosphate-buffered saline (PBS) and blocked for 30 min at room temperature with PBS containing 1% goat serum and 5% fetal bovine serum. The slides were then incubated at room temperature for 1 h with a rabbit anti-Ras antibody (sc-68743; Santa Cruz) diluted 1∶100 in blocking buffer. After washing, the slides were incubated at room temperature for 1 h with an Alexa 594-conjugated goat anti-rabbit IgG antibody (A11012; Invitrogen) diluted 1∶100 in blocking buffer. After washing, the slides were mounted with Prolong® Gold antifade reagent containing DAPI (Invitrogen). Immunofluorescence images were captured using a fluorescence microscope (BX51; Olympus, Tokyo, Japan).

## Results

### Utilization of HrasG12V, SmoM2 and shp53 for the Induction of HCC

**Figure 1:**
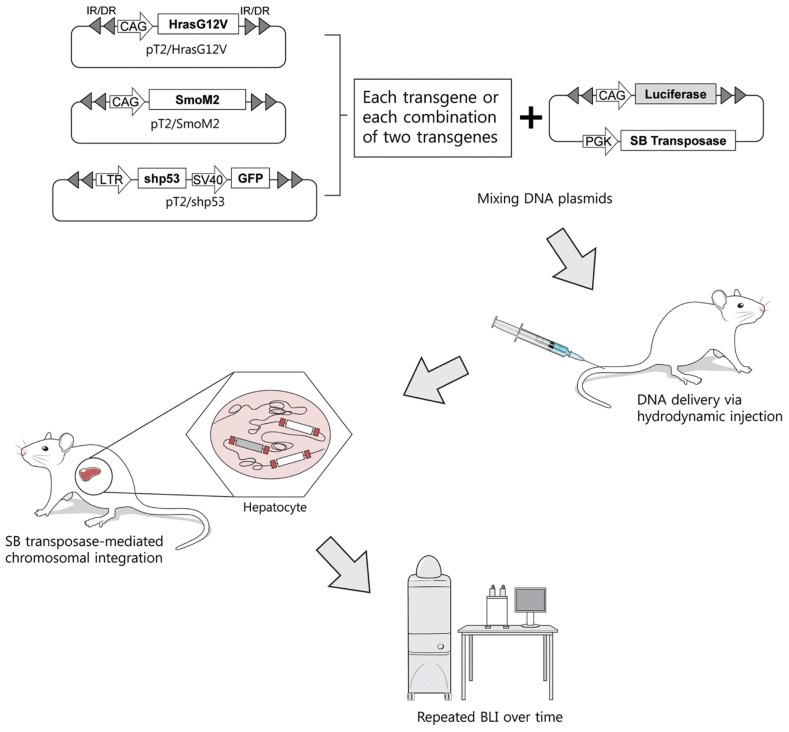
Schematic illustration of the experimental procedure. The transposons were mixed with the plasmids encoding the *Sleeping Beauty* (SB) transposase and were then hydrodynamically delivered to the liver (see the Materials and Methods section). Once in cells, the SB transposase is expressed and binds to the IR/DRs of the transposons. The enzyme subsequently cleaves the transposons at the sites of IR/DRs and integrates them at a new location within the host genome, allowing the transgenes to be stably expressed. Mice are then subjected to repeated bioluminescence imaging over time.

Deregulation of Ras, hedgehog, and p53 signaling pathways is known to be highly related to HCC development in humans. We attempted to develop simple non-germline liver-specific transgenic mouse models in which Ras, hedgehog, and p53 signaling pathways are deregulated, either alone or in combination. Plasmids encoding a constitutively active form of Hras (HrasG12V), a constitutively active form of Smo (SmoM2), and a small hairpin RNA that downregulates TP53 (shp53) were used to deregulate these signaling pathways in this study. The expression cassettes encoding each gene were subsequently placed between two IRs (Fig. 1), rendering them transposable by the *Sleeping Beauty* transposase.

**Figure 2:**
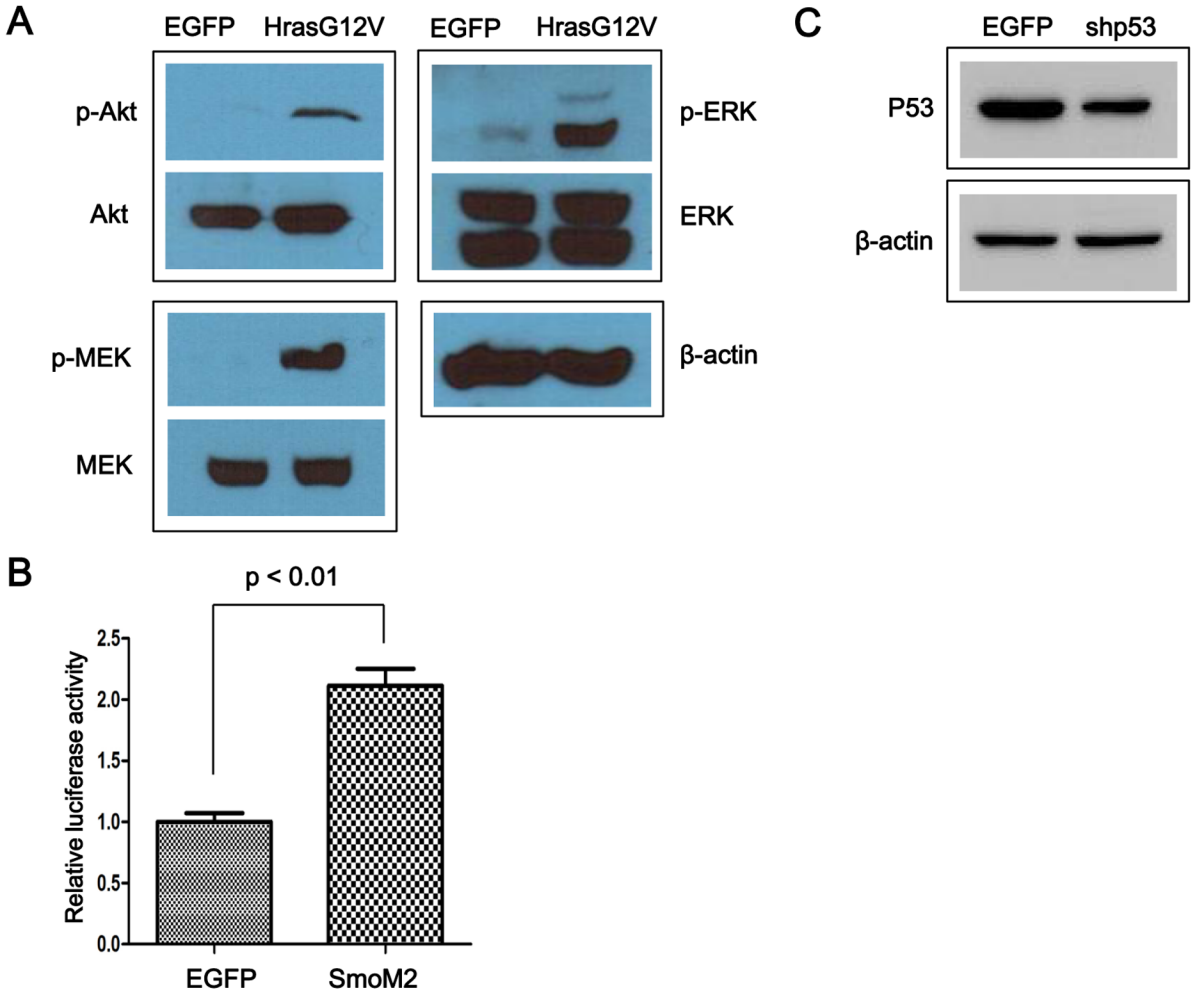
Assessment of the biological functions of HrasG12V, SmoM2, and shp53. (A) Activation of Ras signaling by HrasG12V was confirmed by increased phosphorylation of Akt, ERK, and MEK. (B) Increased activity of Gli transcription factors in response to expression of SmoM2 was detected using a Gli-driven luciferase reporter system. (C) UV-induced expression of p53 was suppressed by shp53 expression. Cells were transfected with either pT2/EGFP (a control) or pT2/shp53 prior to UV irradiation.

To test the expression and biological functions of the genes expressed from the transposons, transient transfection was conducted using NIH3T3 cells. Phosphorylation of the downstream Ras pathway molecules Akt, MEK, and ERK was dramatically increased in cells transfected with pT2/HrasG12V compared to cells transfected with pT2/EGFP, confirming the constitutive activation of Ras signaling by HrasG12V (Fig. 2A). Activation of hedgehog signaling by SmoM2 was confirmed by Gli-luciferase reporter assay. Luciferase expression driven by Gli transcription factors was higher in cells transfected with pT2/SmoM2 compared to cells transfected with pT2/EGFP (p<0.01, see Fig. 2B). Lastly, downregulation of p53 expression by pT2/shp53 was confirmed in an experiment in which p53 expression was induced by UV irradiation (Fig. 2C).

### Hydrodynamic Injection of Oncogene-encoding Transposons and BLI of the Liver

**Figure 3:**
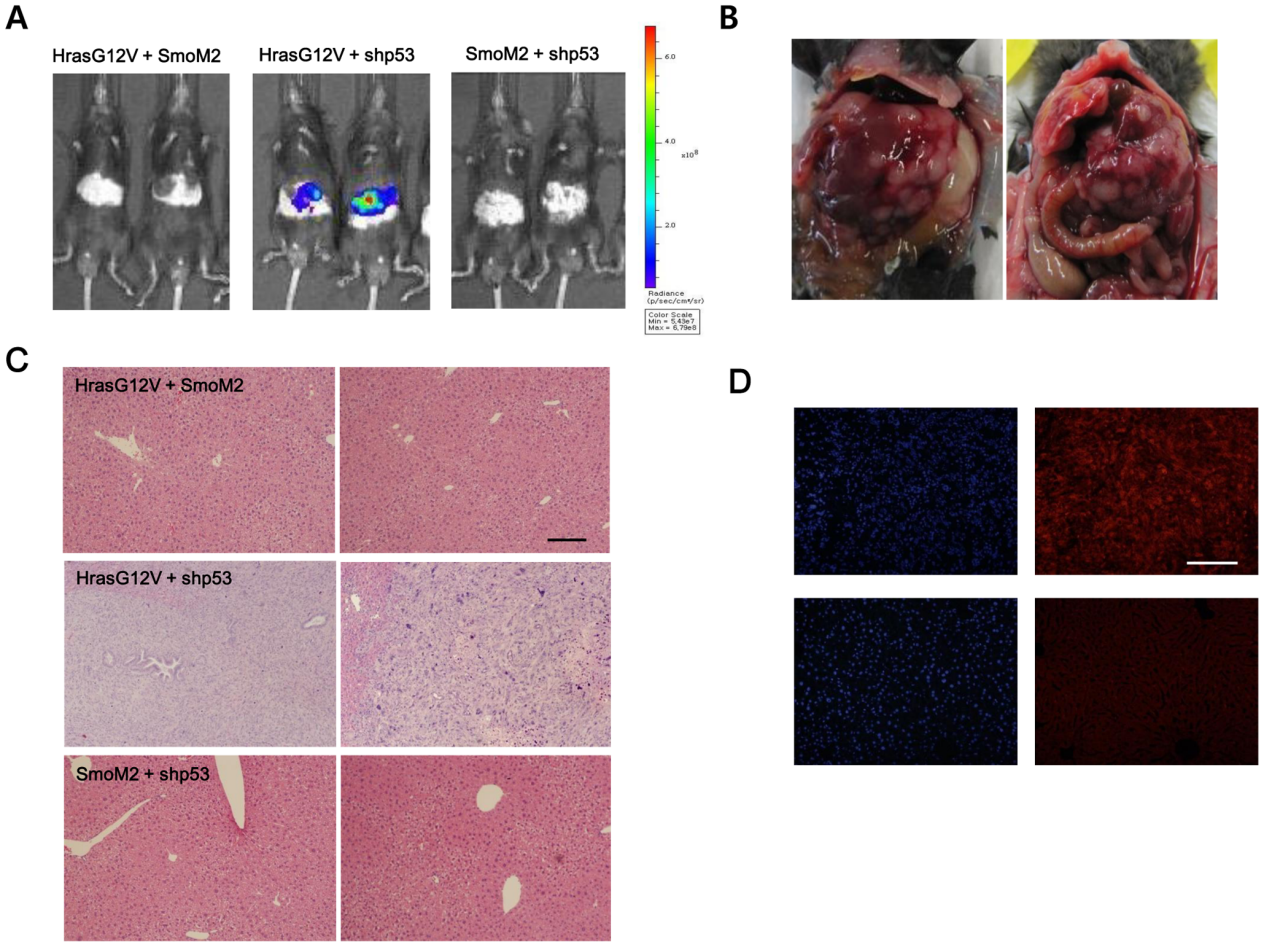
Co-expression of HrasG12V and shp53 induced malignant tumors in the liver. (A) Strong bioluminescence signals were detected in the livers of mice co-expressing HrasG12V and shp53, while other double-transgenic mice revealed no detectable signals. BLI was performed at 4 weeks PHI. (B) Gross morphology of mice of the HrasG12V plus shp53 group at 4 weeks PHI. (C) H&E staining of liver tissues from HrasG12V plus SmoM2 mice (harvested at 30 weeks PHI), HrasG12V plus shp53 mice (harvested at 4 weeks PHI), and SmoM2 plus shp53 mice (harvested at 30 weeks PHI). (D) Immunofluorescence imaging of Ras. Ras expression was detected in tumors induced by HrasG12V and shp53 (upper right panel), while normal liver tissues exhibited background signals (lower right panel). DAPI staining of the same region is shown in the panels to the left. Scale bar, 200 µm.

To generate liver-specific transgenic mouse models, transposons encoding each oncogene were mixed with plasmids encoding the *Sleeping Beauty* transposase (pT2/C-Luc//PGK-SB13) and were then hydrodynamically delivered to the liver (Fig. 1). Once entering a cell, *Sleeping Beauty* transposase can integrate transposons into the genome [24], allowing the transgene to be stably expressed. The plasmid pT2/C-Luc//PGK-SB13 harbors transposons encoding firefly luciferase, chromosomal integration of which allows tumor growth to be monitored by BLI. To generate transgenic mice expressing a combination of two oncogenes, a mixture of pT2/HrasG12V plus pT2/SmoM2, pT2/HrasG12V plus pT2/shp53, or pT2/SmoM2 plus pT2/shp53 was hydrodynamically delivered to the liver together with pT2/C-Luc//PGK-SB13 (Table 1). At 4 days post-hydrodynamic injection (PHI), BLI was performed to confirm successful delivery of plasmid DNA to the liver. Strong BLI signals were observed in all groups; there were no significant differences between groups (see Fig. S1). The strengths of the BLI signals were much lower the following week in all groups, presumably due to the degradation of unintegrated plasmids, consistent with previous reports [25], [26]. At 4 weeks PHI, BLI was performed again and very strong signals were detected in mice in the HrasG12V plus shp53 transgenic group (Fig. 3A). By contrast, only background signals were detected in the HrasG12V plus SmoM2, SmoM2 plus shp53, and single-transgenic groups. The HrasG12V and shp53 double-transgenic mice were subsequently euthanized due to signs of discomfort. For the other groups of mice, BLI experiments were performed every month for up to 7 months PHI. No significant increases in BLI signals were observed in the groups (data not shown).

**Table 1:** Effects of expressed oncogenes on the induction of liver tumors.

Oncogene(s) expressed	% of mice with tumors	Survival (weeks PHI)	Number of nodules
HrasG12V	0 (0/6)	>30	None
SmoM2	0 (0/5)	>30	None
shp53	0 (0/5)	>30	None
HrasG12V, SmoM2	0 (0/8)	>30	None
HrasG12V, shp53	100 (8/8)	<5	Too many to count
SmoM2, shp53	0 (0/8)	>30	None

### Tumor Incidence and Histology

Mice transfected with HrasG12V and shp53 became moribund and exhibited signs of discomfort at about 4 weeks PHI. Livers were harvested from the mice after euthanasia. Tumors were observed in the livers of all mice in this group (Fig. 3B). H&E staining showed highly malignant and undifferentiated tumor cells (Fig. 3C, middle panels). Immunofluorescence imaging confirmed Ras expression in tumors induced by HrasG12V and shp53 (Fig. 3D). No hyperplastic nodules were observed in other double-transgenic groups or the single-transgenic groups when livers were harvested at 7 months PHI. H&E staining also revealed no microscopic nodules in these groups (Fig. 3C, upper and lower panels). Tumor incidence and mouse survival data are shown in Table 1.

### Correlation between Tumor Size and BLI Signal Intensity in Tumors Induced by HrasG12V and shp53

**Figure 4:**
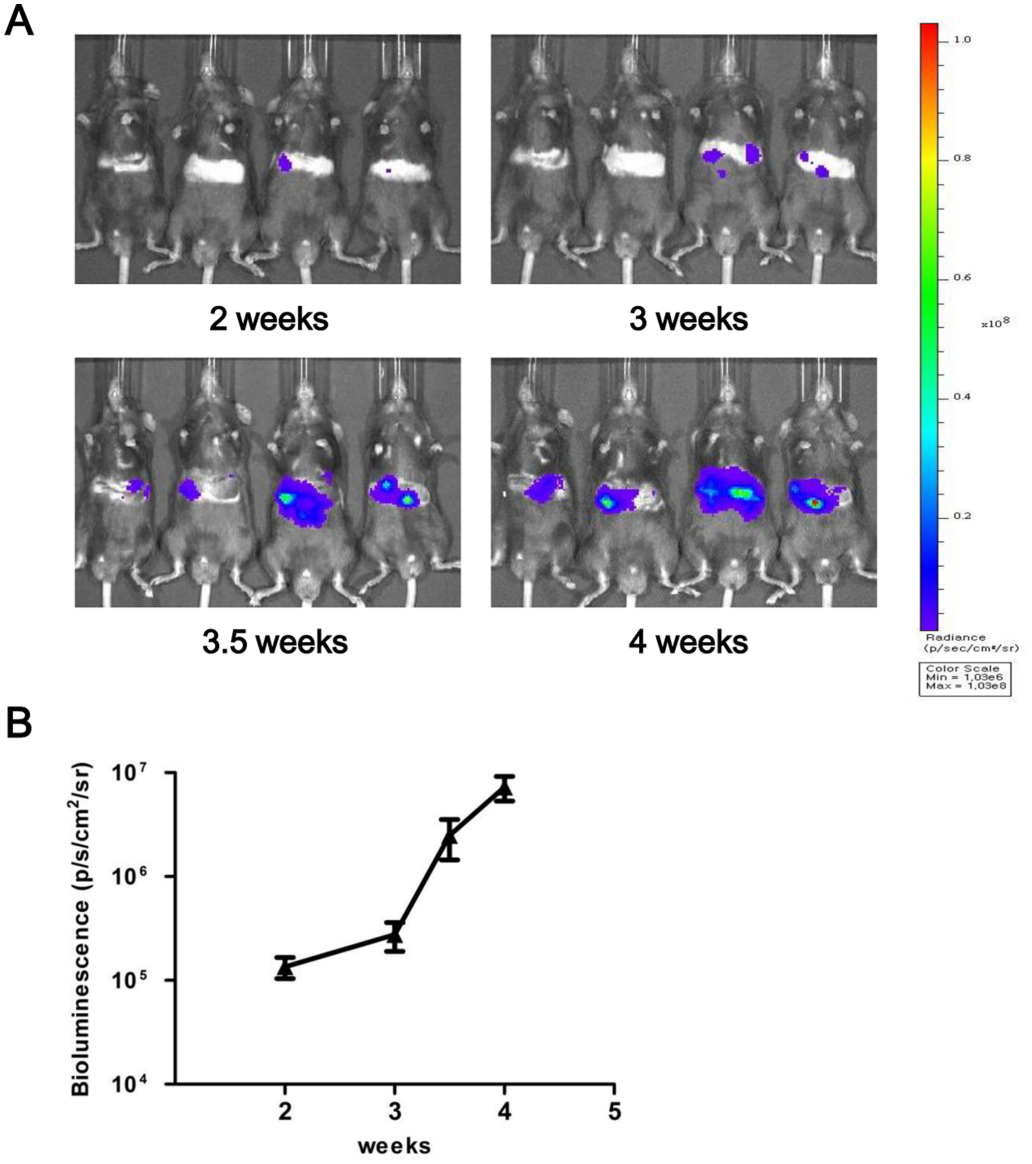
Repeated BLI of the same mice following hydrodynamic injection. (A) Pseudocolor images of tumor growth in the liver following hydrodynamic injection of HrasG12V and shp53. Note the continuous increases in the signals from the abdominal regions of the same mice. (B) Average bioluminescence signals from the abdominal regions of the same mice at the indicated time points PHI.

To test the correlation between tumor size and BLI signal intensity in tumors induced by HrasG12V and shp53, we measured BLI signals at 2, 3, 3.5, and 4 weeks PHI. To completely remove signals from unintegrated transposons encoding firefly luciferase, we started BLI experiments at 2 weeks PHI [25]. Repeated imaging of the same mice over time revealed increases in BLI signals in all mice (Fig. 4A). The increases were most noticeable between 3 and 3.5 weeks PHI. The average bioluminescence signal at each time point is shown in Fig. 4B.

**Figure 5:**
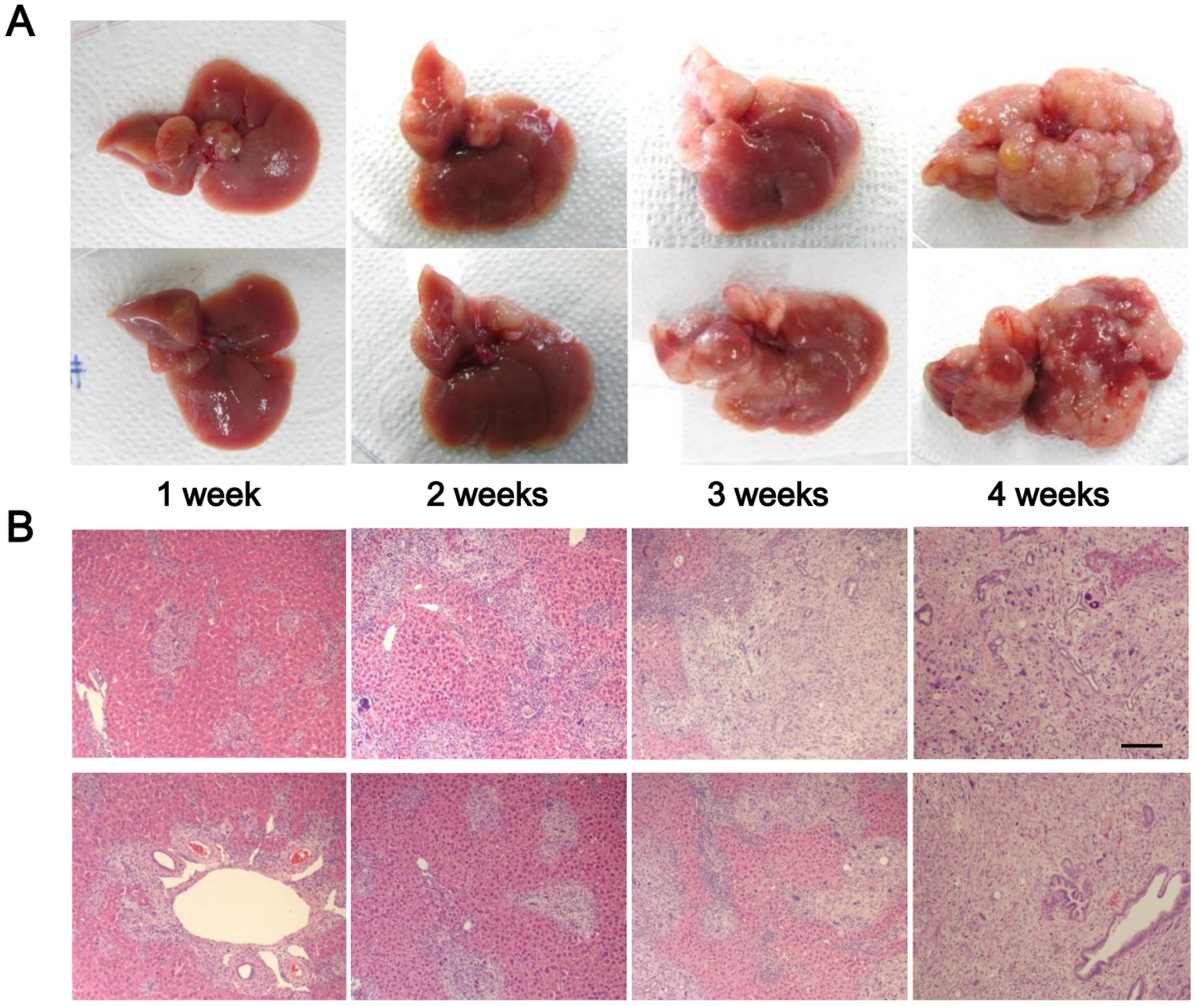
Tumors in the livers of HrasG12V plus shp53 mice following hydrodynamic injection. (A) Gross morphology of livers harvested at 1, 2, 3, and 4 weeks PHI. Note that the tumors grew more rapidly in the right lobes. (B) H&E staining of left caudal lobes harvested at the indicated time points. Scale bar, 200 µm.

To analyze the relationship between BLI signal and tumor burden in the liver, we harvested livers at 1, 2, 3 and 4 weeks PHI and investigated tumor sizes. Based on gross morphology, hyperplastic nodules in the right lobes of the liver were noticed as early as 2 weeks PHI (Fig. 5A). Tumors grew more slowly in the left caudal lobes than in the right lobes. Consistent with the gross morphology, H&E staining of liver tissues in the left caudal lobes revealed increases in tumor size and tissue invasion (Fig. 5B). The results strongly suggest that increases in BLI signals can be a strong indicator of tumor growth in the liver. Thus, tumor growth can be monitored over time in our transgenic model system based on BLI signals, without invasive procedures.

### Oncogenic Collaboration with c-myc

**Figure 6:**
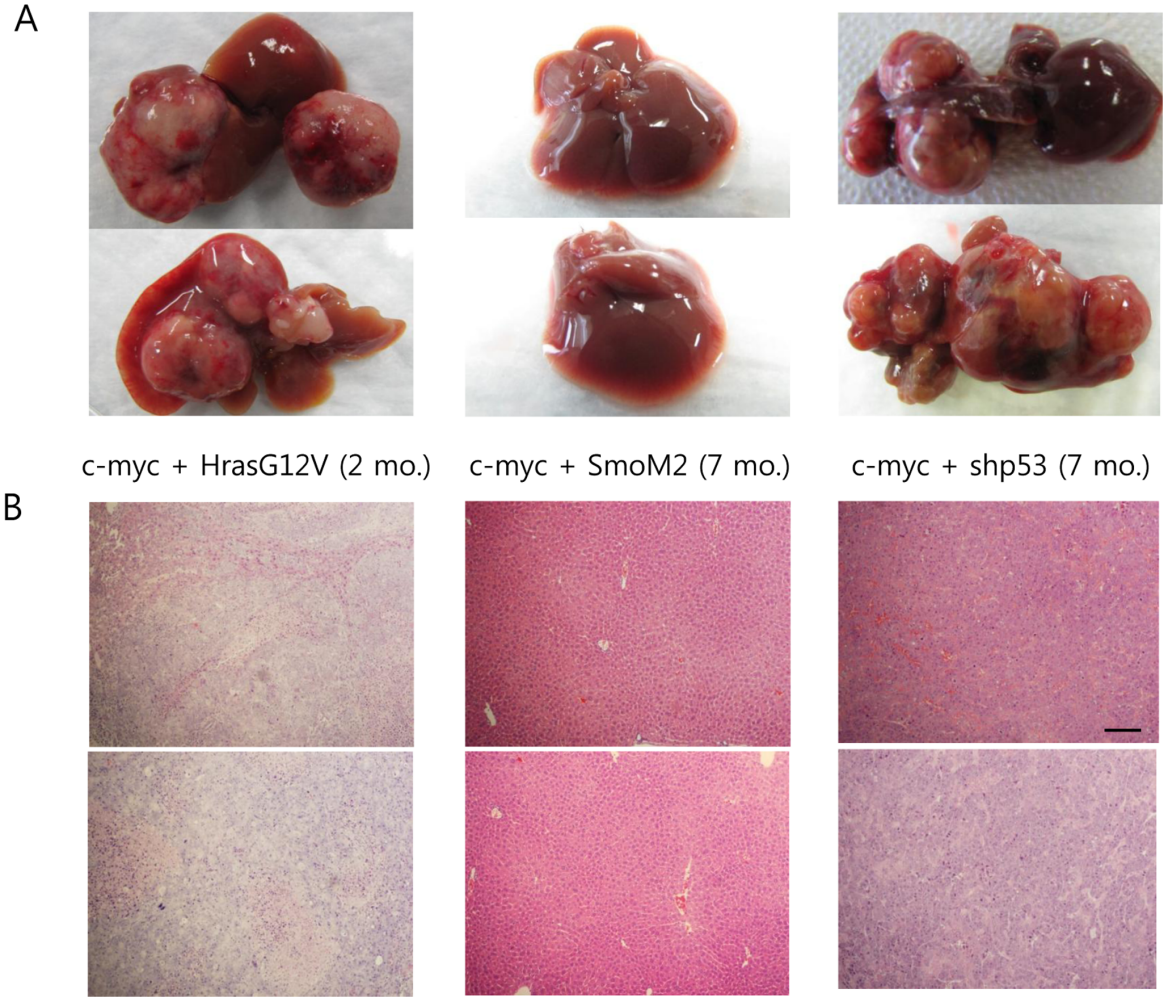
Differential tumorigenic potentials among HrasG12V, SmoM2, and shp53 in the liver. Transposons encoding each oncogene was mixed with c-myc-encoding transposons and then hydrodynamically delivered to the liver. Gross morphology (A) and H&E staining (B) of livers in each group are shown. Note that livers were harvested from c-myc plus HrasG12V mice at 2 months PHI, while livers were harvested in the other groups at 7 months PHI. No hyperplastic nodules were observed in the livers of c-myc plus SmoM2 mice. Scale bar, 200 µm.

Expression of HrasG12V, SmoM2, or shp53 alone failed to induce hepatic tumors in our study. Thus, it is presumed that expression of at least two oncogenes is required to efficiently induce tumors in the liver, as shown in the HrasG12V plus shp53 group. It is not clear why SmoM2 failed to induce hepatic tumors when co-expressed with HrasG12V or shp53 (Fig. 3). One possible explanation is that cooperation of hedgehog signaling with Ras or p53 signaling is not as effective as oncogenic collaboration between Ras and p53 signaling. It is also possible that SmoM2 might be less oncogenic in the liver compared to HrasG12V or shp53. In this regard, we tested the hepatocarcinogenic potential of each oncogene in the liver where c-myc is overexpressed. The c-myc is known to play an important role in hepatocarcinogenesis and often overexpressed in human liver cancers. Transposons encoding c-myc (referred to as “pT2/c-myc”) was mixed with each of pT2/HrasG12V, pT2/SmoM2, and pT2/shp53 and then each combination of oncogenes was hydrodynamically delivered to the liver together with transposase-encoding plasmids (see Method S1 for the detailed method). Tumors were found in the c-myc plus HrasG12V group as early as at 2 months PHI and in the c-myc plus shp53 group at about 7 months PHI (Figure 6). However, no hyperplastic nodules were observed until 7 months PHI in the c-myc plus SmoM2 group. This data also supports the idea that SmoM2 might be less oncogenic than HrasG12V or shp53 in the liver.

### Removing the Transposase Reduces the Numbers of Hyperplastic Nodules

**Figure 7:**
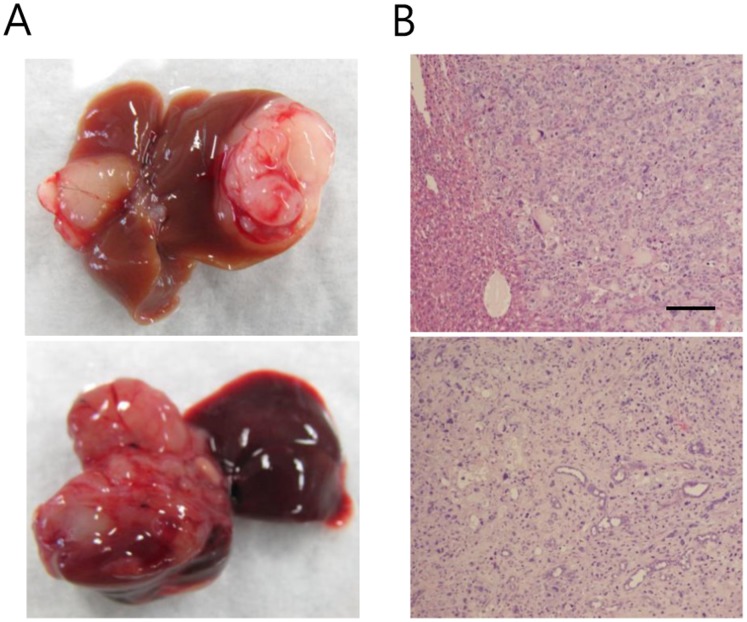
Tumors induced by transposon vectors encoding HrasG12V and shp53 without the *Sleeping Beauty* transposase. (A) A few large hyperplastic nodules were observed at 3 months PHI. (B) H&E staining of the tumors shown in (A). Scale bar, 200 µm.

The liver tumors induced by HrasG12V and shp53 exhibited numerous hyperplastic nodules. For some applications, such as efficacy testing of anti-cancer drugs, it might be desirable to use a tumor model with fewer hyperplastic nodules. For this reason, we transfected the liver with a mixture of pT2/HrasG12V and pT2/shp53, without plasmids encoding *Sleeping Beauty* transposase. Under these conditions, chromosomal integration of the transgenes solely relies on a spontaneous process [27]. Two of five mice showed signs of discomfort at 3 months PHI and a few large hyperplastic nodules were found in their livers (Fig. 7A and B). Thus, removing the *Sleeping Beauty* transposase greatly reduced the numbers of hyperplastic nodules. The other three mice, however, did not show visible hyperplastic nodules when their livers were harvested at 9 months PHI. This is a potential problem in preclinical testing of anti-cancer drugs. Rather than completely removing plasmids encoding *Sleeping Beauty* transposase, using a minimal dose of the plasmids might be an option to induce tumors in all mice while still keeping the numbers of tumor nodules low.

## Discussion

In this study, we presented a simplified methodology with which the tumorigenic potential of individual genes and combinations of genes can easily be tested in the liver *in vivo*. First, we generated non-germline liver-specific transgenic mouse models more quickly and cheaply using hydrodynamics-based transfection and the *Sleeping Beauty* transposon system. Second, we utilized firefly luciferase as a reporter, allowing tumor growth in the liver to be easily monitored via BLI without an invasive procedure. Application of the methodology is expected to accelerate and facilitate *in vivo* studies of the oncogenic potential of cancer-related genes in the liver. Although the methodology is considered versatile and cost-effective in generating transgenic models for liver cancer and monitoring tumor growth, there are some potential disadvantages of the method. Because transgenic mouse developed by hydrodynamic injection cannot transmit transgenes to offspring, DNA injection should be performed for each tumorigenic study. Furthermore, due to a high volume of solution injected rapidly via the tail vein, liver might experience a mechanical injury after DNA injection although hydrodynamic injection is generally considered to cause little harm [28].

Using this strategy, we tested the oncogenic potential of HrasG12V, SmoM2, and shp53 in the liver. Mice with simultaneous expression of HrasG12V and shp53 in the liver exhibited very strong BLI signals in the abdominal area. Consistent with the BLI data, gross morphology revealed rapidly induced tumors in the liver with numerous hyperplastic nodules. Tumors in this group were also highly malignant and poorly differentiated (Fig. 3C). Expression of either HrasG12V or shp53 alone, however, failed to induce hepatic tumors in our study. Thus, it is presumed that both proliferation and anti-apoptotic signals are required to efficiently induce tumors in the liver. Simultaneous expression of HrasG12V plus SmoM2 or SmoM2 plus shp53 also failed to induce tumors in the liver. The reason why tumors were not observed in the other double transgenic mice is unclear. SmoM2 induces activation of hedgehog signaling, leading to cellular proliferation in many tissues [9], [10]. One possible explanation is that SmoM2 might be less oncogenic in the liver compared to HrasG12V or shp53. We further tested the hepatocarcinogenic potential of SmoM2 via co-expression with c-myc. No hyperplastic nodules were observed in the livers of c-myc plus SmoM2 mice until 7 months PHI while tumors were observed both in the c-myc plus HrasG12V and c-myc plus shp53 groups. This also suggests that SmoM2 is less oncogenic than HrasG12V or shp53 in the liver. Although SmoM2 failed to cooperate with HrasG12V, shp53, and c-myc in inducing hepatic tumors, however, we cannot rule out the possibility that SmoM2 could induce hepatic tumors via oncogenic collaboration with other types of oncogenes. More extensive studies should be performed to address this issue.

Due to the difficulty of accessing the liver, liver tumor sizes are hard to measure without invasive surgery or killing the animal. Imaging techniques such as micro-computed tomography and micro-positron emission tomography have been developed to detect tumor lesions and to quantify tumor load in small animals [29]. Although recent years have seen refinements and advances in imaging techniques, they are still not easily accessible to many researchers due to the high cost of imaging instruments and technical difficulties. Optical imaging techniques such as fluorescence imaging and BLI are, however, cost effective and relatively easy to use [30], [31]. Firefly luciferase was successfully utilized in our study to monitor changes in tumor sizes *in vivo*. Increases in BLI signals were well correlated with actual tumor growth in the liver in our transgenic mouse model, confirming the versatility of BLI in monitoring tumor growth without an invasive procedure.

One important application of our model system is preclinical testing of therapeutic drugs for liver cancer. Retardation of tumor growth or reduction of tumor size could be effectively monitored by repeated BLI over time following drug administration. Currently, we are applying the transgenic model to study the anti-cancer effects of Akt inhibitors and dietary intervention (manuscript in preparation). It is expected that the applicability of the transgenic liver cancer model will broaden because of the ease of development of the tumor model and efficient *in vivo* imaging of tumor growth.

## Supporting Information

Figure S1 **Bioluminescence imaging performed at 4 days post hydrodynamic injection.** Strong bioluminescence signals were observed from the livers of all mice, confirming successful delivery of transgenes to the liver. No significant differences in bioluminescence signals were found among the double transgenic groups. Similar results were obtained from the single transgenic groups (data not shown).
(TIF)

Method S1 **Hydrodynamic injection of c-myc-encoding transposons.**
(DOC)
