# Supplementary figures and images for: Investigation of Oncogenic Cooperation in Simple Liver-Specific Transgenic Mouse Models Using Noninvasive In Vivo Imaging
Source: PLoS One. 2013 Mar 28;8(3):e59869. doi: 10.1371/journal.pone.0059869 (PMC3610734; doi:10.1371/journal.pone.0059869)

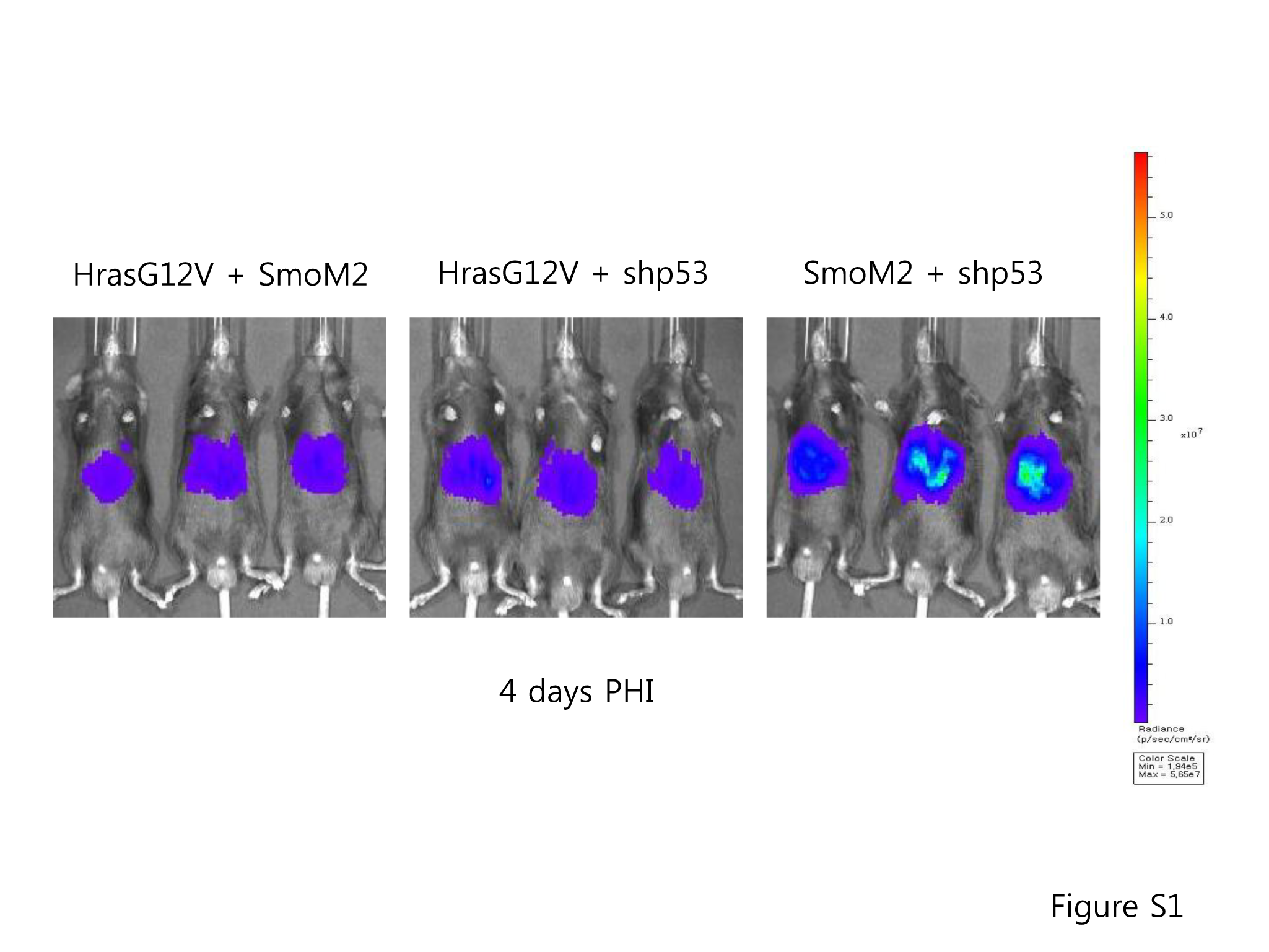

Supplement: Figure S1 — Bioluminescence imaging performed at 4 days post hydrodynamic injection. Strong bioluminescence signals were observed from the livers of all mice, confirming successful delivery of transgenes to the liver. No significant differences in bioluminescence signals were found among the double transgenic groups. Similar results were obtained from the single transgenic groups (data not shown). (TIF) [file pone.0059869.s001.tif]
