# Supplementary material for: Investigation of Oncogenic Cooperation in Simple Liver-Specific Transgenic Mouse Models Using Noninvasive In Vivo Imaging
Source: PLoS One. 2013 Mar 28;8(3):e59869. doi: 10.1371/journal.pone.0059869 (PMC3610734; doi:10.1371/journal.pone.0059869)
Supplement: Method S1 — Hydrodynamic injection of c-myc-encoding transposons. (DOC) [file pone.0059869.s002.doc]

Method S1

**Hydrodynamic injection of c-myc-encoding transposons**

A cDNA encoding the wild-type murine c-myc was PCR amplified from pCX-cMyc (plasmid #19772; Addgene) as a template using the following primer pair: forward, 5’- CGA ATT CGC CAC CAT GCC CCT CAA CGT GAA CTT C -3’; and reverse, 5’- CGA ATT CTT ATG CAC CAG AGT TTC GAA GCT G -3’. The amplified products were then digested with *Eco*RI and cloned into pT2/EGFP following digestion of the plasmid with the same restriction enzyme to remove the EGFP cDNA. The resulting plasmid is referred to as pT2/c-myc, harboring the c-myc gene flanked by two inverted terminal repeats (IRs). 14 μg of pT2/c-myc (7.4 kb) was mixed with each of the following plasmids, 12.5 μg of pT2/HrasG12V (6.6 kb), 16 μg of pT2/ SmoM2 (8.4 kb), and 14 μg of pT2/shp53 (7.4 kb). The molar amount of each transposon plasmid used was the same, regardless of the types of transposons. Each combination of transposons was then mixed with 9 μg of pPGK-SB13 (a generous gift from Dr. David Largaespada at the University of Minnesota). After mixing transposons with the transposase-encoding plasmids, DNA was suspended in 2 ml of lactated Ringer’s solution and was then injected into the lateral tail veins of male 6- to 7-week-old C57BL/6 mice (0.1 ml/g body weight) in less than 7 sec.
